# Supplementary material for: Individual traits and experiences predict the content of dreams
Source: Commun Psychol. 2026 Apr 28;4:69. doi: 10.1038/s44271-026-00447-2 (PMC13124594; doi:10.1038/s44271-026-00447-2)
Supplement: Supplementary file 2 — Supplemental Material [file 44271_2026_447_MOESM2_ESM.pdf]

SUPPLEMENTARY MATERIALS

Individual Traits and Experiences Predict the Content of Dreams

Valentina Elce [1*], Giorgia Bontempi [1], Serena Scarpelli [2], Bianca Pedreschi [1], Pietro Pietrini [1], Luigi De Gennaro [2], Michele Bellesi [3], Giulio Bernardi [1*†], Giacomo Handjaras [1*†]

[1] MoMiLab Research Unit, IMT School for Advanced Studies Lucca, Lucca, Italy
[2] Department of Psychology, Sapienza University of Rome, Rome, Italy
[3] School of Biosciences and Veterinary Medicine, University of Camerino, Camerino, Italy

† These authors contributed equally to this work

*** Correspondence:**
Valentina Elce
IMT School for Advanced Studies Lucca
Piazza San Francesco, 19
55100 Lucca - Italy
email: valentina.elce@imtlucca.it

Giulio Bernardi
IMT School for Advanced Studies Lucca
Piazza San Francesco, 19
55100 Lucca - Italy
email: giulio.bernardi@imtlucca.it

Giacomo Handjaras
IMT School for Advanced Studies Lucca
Piazza San Francesco, 19
55100 Lucca - Italy
email: giacomo.handjaras@imtlucca.it

Supplementary text

Validating the AI scoring of dimensions

In this initial control experiment, we evaluated the reliability of automatic techniques for quantifying semantic content by comparing computational linguistics measurements with manual dream content scoring performed by four trained external human raters.

Participants and scoring procedure

4 expert external human raters (3 females, mean age: 31 ± 5.35 y, all postgraduates with an advanced training in the field of sleep research) were enrolled to score a subsample of the *main* dataset including 823 dream reports provided by 86 participants (50 female participants, 36 male participants, mean age: 34.68 ± 11.03), based on the 16 semantic dimensions described above. In particular, the raters were asked, for each report, to answer 16 questions on a Likert scale from 1 to 9. Each question regarded a semantic dimension.

The task was autonomously performed by the scorers on their computers through a custom-made graphical interface that was implemented and presented in MATLAB (The Mathworks Inc., 2022a). Before performing the task, the scorers were provided with written instructions on how to interpret the dimension definitions and answer the questions. The interface automatically saved the scored data at the end of each session.

Each dream report appeared in a white box in the center of the interface. The reports were displayed in random order across the scorers. Below the report, only one question at a time was displayed. Raters scored each semantic dimension by providing their answers using a slider along a continuous scale (Likert from 1 to 9), with the possibility to select values between the displayed labels. Once they answered one question, scorers were asked to press the button "forward" ("*avanti*") at the bottom of the screen for switching to the next question. At the end of all 16 questions, the following dream report appeared. At the top right of the screen, scorers could also see a counter of the dreams scored within the current session. The median time needed by the scorers to complete the 16 questions for one dream was on average 2'18" (range 1'28" - 2'54"), for a total of about 32 hours per rater.

Measuring the alignment between external human observers and AIs

We first assessed the agreement between the dimensional scores provided by the external human raters. To account for participant variability, we measured Spearman's correlation coefficients among raters for each of the 86 participants. For each pairing of raters, we calculated the median of the correlation coefficients across participants to obtain an overall group-level measure. Additionally, we measured the noise-ceiling boundaries by evaluating the correlation of each rater's scores with the average (median) scores across all four raters to obtain an upper bound, and the correlation of each rater's scores with the average (median) scores from the other three raters to estimate the lower bound [53]. Results of the agreement among external human raters during the evaluation of the 16 semantic dimensions are shown in Supplementary Fig. S1A.

Similarly to the previous approach, we assessed the agreement between the external human raters and each of the three AIs (i.e., LLaMA 3, ChatGPT-4, and ChatGPT-4 Turbo) by estimating Spearman's ρ between the raters and the AIs for each participant independently. We also estimated the correlation between the median ratings across raters and the median scores across AIs, with the results reported in Supplementary Fig. S1B.

Overall, these results indicated that the scores provided by the three AIs, particularly when combined, showed a strong similarity with those obtained from external human raters.

Evaluating the Alignment of Dreamers' Subjective Ratings with Human and AI observers

The second control experiment was a dream diary study, in which participants recorded their dream experiences for 15 days immediately upon waking and evaluated their own dream content. This study aimed to determine the extent to which external human and artificial rating could reflect the semantic characterization of the individuals' subjective experience.

Participants

In this control experiment, 10 Italian native language speakers (6 female participants, 4 male participants, mean ± std, age 30.18 ± 4.7 yrs; range 25-41 y) were recruited. Similar to the paradigm described above, we only recruited individuals with regular sleep/wake patterns, six to eight hours of sleep per night, and no diagnosis of sleep-related disorders or of any other pathological condition that might have compromised their sleep.

Recording of verbal dream reports, sleep patterns, and subjective ratings

In this study, participants were provided with an actigraph and were required to complete a 15-day dream diary only. Volunteers were provided with the same instructions used in the *main* dataset and described above. Here, participants recorded and sent their sleep conscious experience reports to an experimenter via vocal messages.

Immediately after completing the recording, participants were asked to fill out a questionnaire consisting of 23 items divided into two sections. In the first section, if participants were able to recall the content of their dreams upon awakening, they responded to 17 questions on a 9-point Likert scale. These questions assessed the semantic dimensions of their dream experience, as described above, as well as their confidence in the completeness and accuracy of their recollections. The second section of the questionnaire focused on participants' current mood, level of tiredness, and the perceived quality of their nocturnal sleep, all rated on a 9-point Likert scale. Additionally, they provided information on their falling asleep and awakening times and indicated whether their awakening was spontaneous or induced by external factors such as the alarm, the bed partner or other noises. If participants awoke with the feeling of having dreamt but were unable to recall details of the experience, or if they felt they had not dreamt at all, they were instructed to complete only the second section of the questionnaire, reporting on their mood, tiredness, sleep quality, sleep duration, and the nature of their awakening.

Notably, during the recruitment phase, participants were provided with written instructions regarding the questions they were asked to answer and including the specific definitions of each semantic dimension, which were based on the prompting input to the AI models.

Finally, from a minimum of 30 to a maximum of 40 days after completing the task, participants were contacted again for a follow-up session. They were asked to read the reports of the contentful dream experiences they recorded during the 15-day dream diary protocol and, for each report, to answer the 16 questions regarding the semantic dimensions and assess, on a 9-point Likert Scale, how vivid was their memory of the dream experience. Of note, before the follow-up, reports of sleep conscious experience were treated up to the second-level preprocessing used in the main study and described above. This follow-up task was submitted online and the reports were administered in random order. Overall, in this control experiment we gathered 64 dream reports (reports per participant: 6.4±3.3, min:2, max:12).

Measuring the alignment between subjective ratings, human and AIs observers

We first assessed the agreement between the dimensional scores provided by dreamers immediately after awakening and after the 30-day follow-up. Similar to the previous approach, Spearman's correlation coefficient was calculated for each participant, and then the median estimate for each dimension was computed. Results of the agreement among dreamers during the evaluation of the 16 semantic dimensions are shown in Supplementary Fig. S2A. We then assessed the agreement between dreamers and each of the three AIs (i.e., LLaMA 3, ChatGPT-4, and ChatGPT-4 Turbo) and their combination, using the same approach used above. Results are reported in Supplementary Fig. S2B.

Supplementary Figures

Figure S1

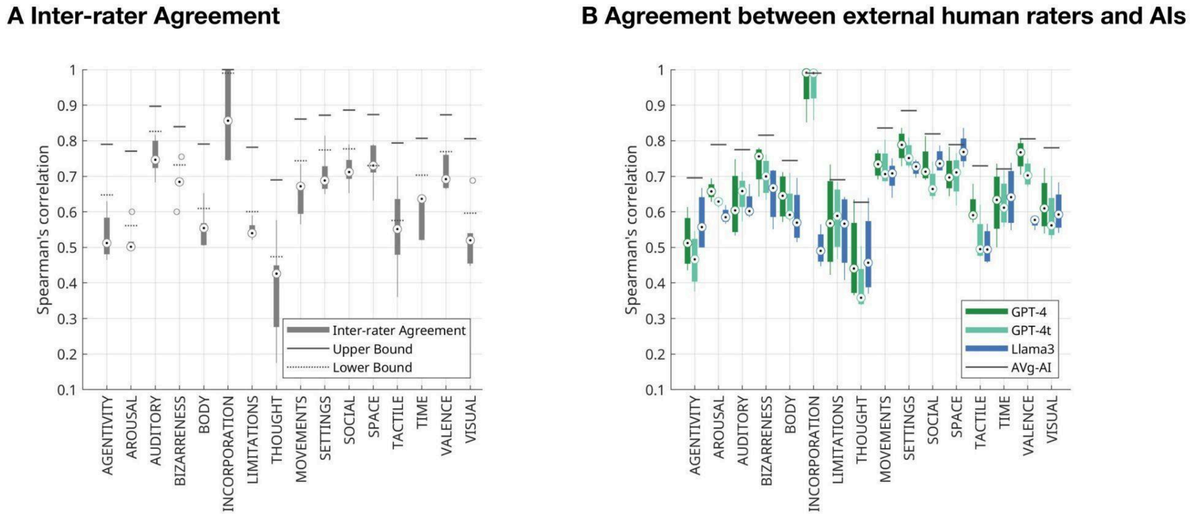

Fig. S1. Agreement among external human raters and AIs in the evaluation of the 16 semantic dimensions. *(**A**) The boxplots represent the Spearman correlation coefficients among raters for each semantic dimension. The noise-ceiling boundaries are also displayed. The upper bound was computed by evaluating the correlation of each rater's scores with the average (median) scores across all four raters. The lower bound was estimated as the correlation of each rater's scores with the average (median) scores from the other three raters. The average agreement was* ρ*=0.626±0.115, with a minimum* ρ*=0.427 for thoughts, and a maximum of* ρ*=0.856 for incorporation. (**B**) The boxplots represent the Spearman's correlation coefficient between the external human raters and each of the three AIs (i.e., LLaMA 3, ChatGPT-4, and ChatGPT-4 Turbo) for each semantic dimension. The correlation between the median ratings across raters and the median scores across AIs (AVg-AI) is also displayed with a black continuous line with an average agreement of* ρ*=0.781±0.085 (minimum* ρ*=0.627 for thoughts, maximum of* ρ*=0.990 for incorporation). The scores from AVg–AI were employed for all the statistical analyses described in the main text.*

Figure S2

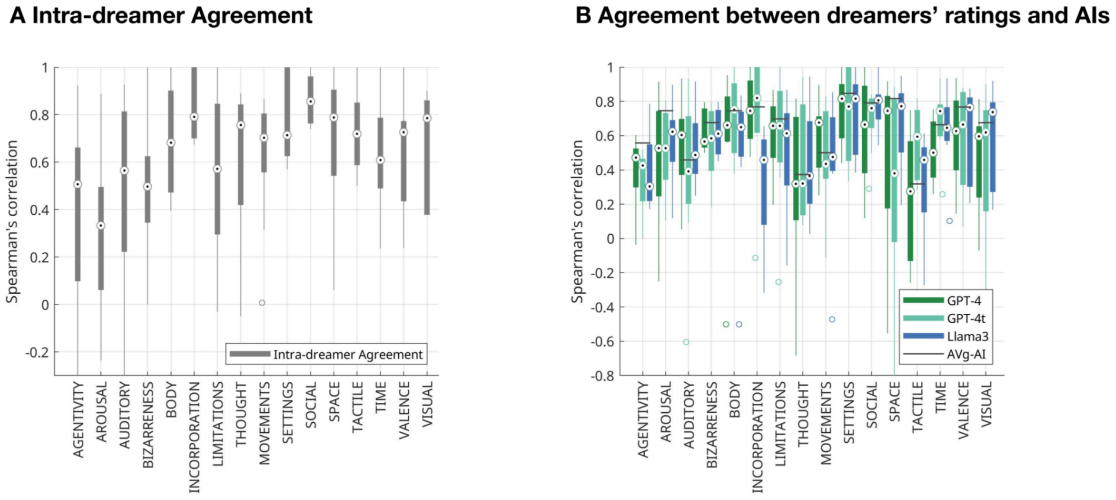

Fig. S2. Agreement between dreamers and among dreamers and AIs in the evaluation of the 16 semantic dimensions. *(**A**) Spearman's correlation coefficient between the scores provided by dreamers immediately after awakening and after the 30-day follow-up for each semantic dimension. The average agreement was* $\rho=0.663\pm0.138$*, with a minimum* $\rho=0.333$ *for arousal, and a maximum of* $\rho=0.856$ *for social. (**B**) Spearman's correlation coefficient between dreamers and each of the three AIs (i.e., LLaMA 3, ChatGPT-4, and ChatGPT-4 Turbo) for each semantic dimension. The correlation coefficient between the median ratings across dreamers and the median scores across AIs (AVg-AI) is also displayed with a black continuous line with an agreement of* $\rho=0.650\pm0.161$ *(minimum* $\rho=0.319$ *for tactile, maximum of* $\rho=0.847$ *for settings). The scores from AVg–AI were employed for all the statistical analyses described in the main text.*

Figure S3

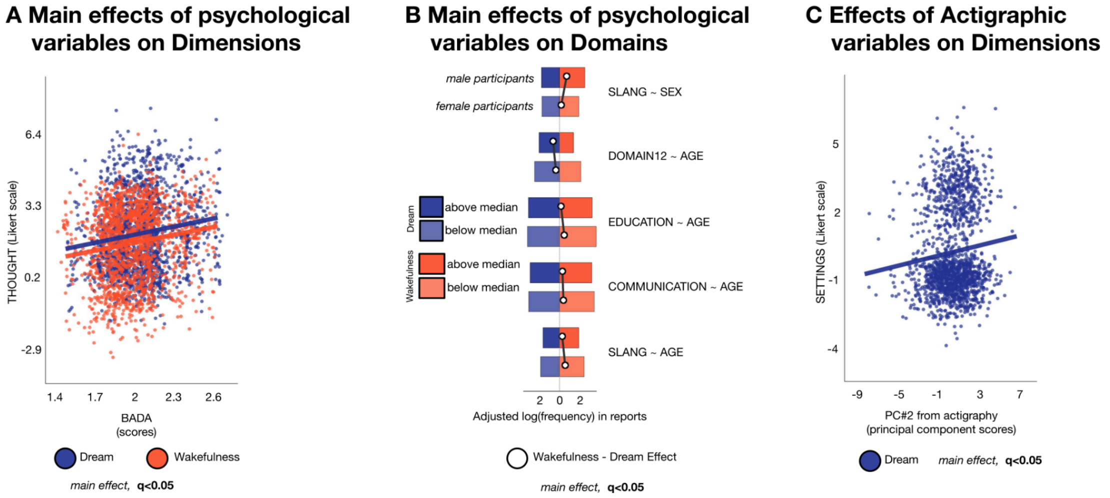

Fig. S3. Effect of individual variables on dream and wakefulness report content. *Significant effects of individual variables on semantic dimensions and domains (GLME model using psychological variables and their interaction with vigilance states as regressors of interest and age, sex, education level, and the BADA score as covariates; q<0.05, FDR correction). (**A**) The scatter plot shows the significant effect of individual verbosity (BADA scores) on the description of thoughts and metacognitive processes (thought dimension) in distinct vigilance states. Each dot represents a different report, with dream reports shown in blue and wakefulness reports displayed in red. Trend lines are also drawn for wakefulness (red) and dream (blue) reports. A slight jittering of points is applied to enhance visualization. (**B**) The bar plot shows significant effects of individual variables on lexical domains. White dots indicate the frequency differences between wakefulness (red) and dream (blue) reports. Bars displayed in darker and lighter colors respectively indicate the reports with the highest (above median) and lowest (below median) values of each predictor. The plot evidences an increased use of slang in wakefulness reports provided by younger male participants, whereas older volunteers showed lower lexical references to education, communication dynamics and domain12. (**C**) The scatterplot shows the significant effect of actigraphy-based PC2 (long light sleep) on setting shifts in dream reports (blue dots); the trend line is also drawn in blue. A slight jittering of points is applied to enhance visualization.*

Figure S4

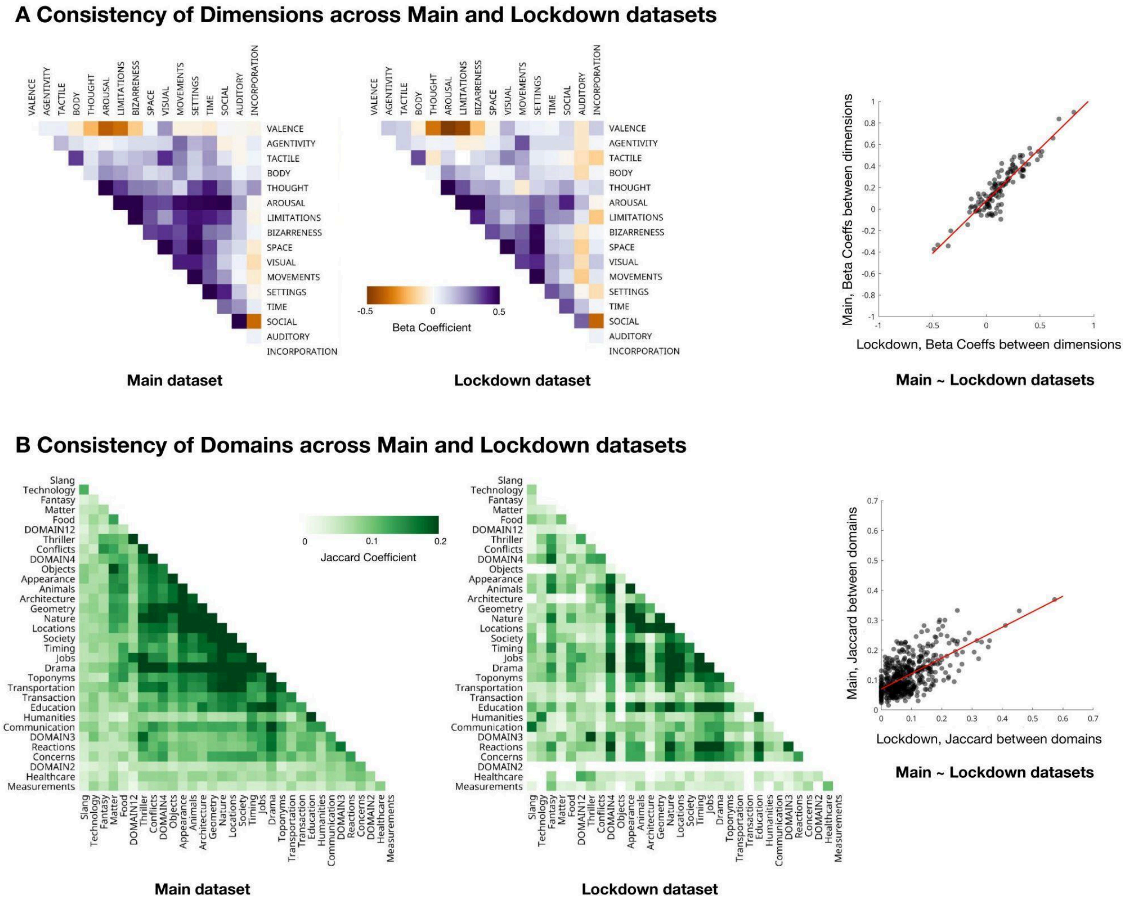

Fig. S4. Consistency of semantic dimensions and lexical domains across main and lockdown datasets. *Beta coefficients for dimensions and Jaccard indices for domains are reported (computed through Generalized linear mixed-effect -GLME- model, using age, sex, education level, and verbosity (BADA score) as covariates of no interest; q<0.05, False discovery Rate -FDR- correction), along with the similarity between dimensions and domains across the two datasets. (**A**) Matrices display the beta coefficient in dream reports only among semantic dimensions in main (left) and lockdown (right) datasets. The scatter plot shows the beta coefficients in the main and lockdown dataset. The agreement between dimension across the two dataset was* $\rho=0.873$, $p<0.00001$. *(**B**) Matrices report the Jaccard index to quantify the overlap of lexical domains in main (left) and lockdown (right) datasets. Empty cells indicate non-significant associations. The scatter plot shows the Jaccard indices in the main and lockdown dataset. The scatter plot shows the beta coefficients in the main and lockdown dataset. The agreement between domains across the two dataset was* $\rho=0.554$, $p<0.00001$.

Figure S5

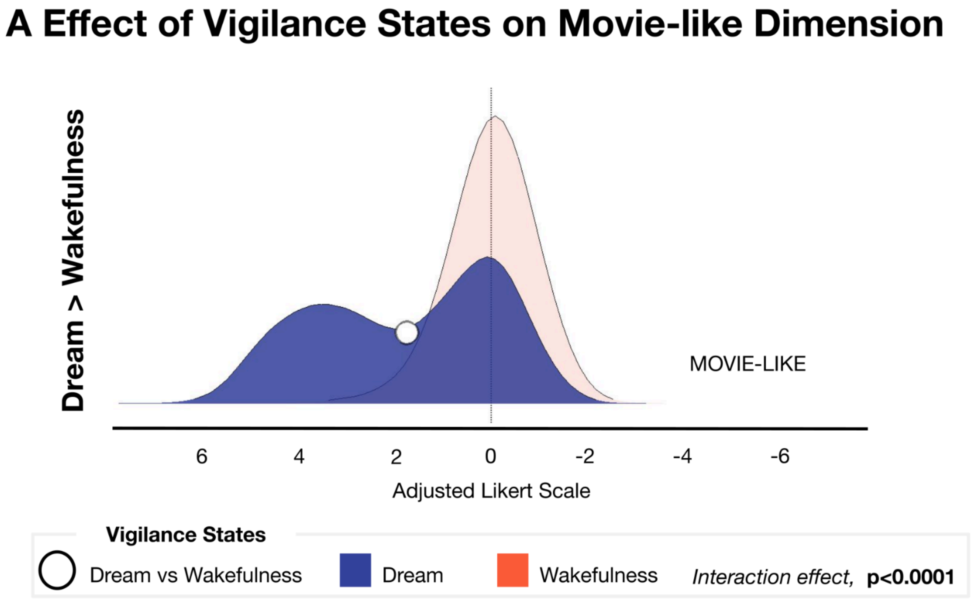

Fig. S5. Movie-like differences between vigilance states, i.e., dream versus wakefulness reports. *We conducted an additional complementary analysis explicitly testing whether dream reports are judged as more movie- or screenplay-like than waking reports. Using an approach parallel to our hypothesis-driven semantic dimension analyses, we prompted LLMs to rate each report on a 1–9 scale for similarity to a movie or theatrical plot (full prompt below). The plot illustrates the distribution of the semantic dimension (rated on a 1–9 Likert scale) for wakefulness (light red) and dream (blue) reports. Likert scores are reported as adjusted for age, sex, education level, and BADA score. Dream reports received significantly higher movie-likeness scores than waking reports (GLME, permutation test, n = 5000; movie-like coefficient = 1.7337, CI: 1.6356 1.8318, Cohen's d =* 1.37, *p <* *0.0001). White dots indicate the differences in scores between dream and wakefulness reports. The prompt used for the three AIs was: "Evaluate how much the Italian text resembles a movie plot, a screenplay or a story that could be turned into a theatrical play of any genre on a scale from 1 to 9. By movie plot we mean a text that describes characters, conflicts, events, and narrative progression. By screenplay we mean a text that contains scene descriptions, actions, and character dialogue. Assign 1 if the text does not resemble any kind of movie-like story. Assign 5 if it moderately resembles a movie-like story. Assign 9 if it strongly resembles a movie-like story suitable for adaptation into a screenplay. Do not evaluate technical screenplay formatting. Short or concise texts should not be penalized. Focus on the story's cinematic potential, not its length or the presence of dialogue. Answer only with a number. Here is the text in Italian:".*

Supplementary Tables

Table S1

DIMENSIONS	PROMPTING
AGENTIVITY	"Evaluate how much the narrator of an Italian text is the agent of the actions described on a scale from 1 to 9. The scoring does not concern whether the valence of the actions is positive or negative. With score 1 you need to score text where the narrator is extremely passive and undergoes the actions performed by others. With score 5 you need to score text where the narrator is neither passive or active or does not take part into the actions described. With score 9 you need to score text where the narrator is extremely active and perform the actions. Answer only with a number. Here is the text in Italian: "
AROUSAL	"Evaluate the emotional intensity of an Italian text on a scale from 1 to 9. The scoring is not related to the emotional valence. The score only concerns the emotional strength of the narration. With score 1 you need to score text not at all emotionally intense. With score 5 you need to score text moderately intense. With score 9 you need to score text extremely intense. Answer only with a number. Here is the text in Italian: "
AUDITORY	"Evaluate how much an Italian text describes auditory experiences on a scale from 1 to 9. You should evaluate how much the situations include talking, hearing, listening and any details that can only be perceived either by the narrator or other characters through the hearing, such as voices, noise and music. With score 1 you need to score text without any semantic relatedness to auditory experiences. With score 5 you need to score text with a moderate semantic relatedness to auditory experiences. With score 9 you need to score text with extreme semantic relatedness to auditory experiences. Answer only with a number. Here is the text in Italian: "
BIZARRENESS	"Evaluate the bizarreness of an Italian text on a scale from 1 to 9. You should evaluate how much the situations described in the text are strange or unnatural according to your specific experience. With score 1 you need to score text describing completely normal and common situations. With score 5 you need to score text describing partially strange or unnatural situations. With score 9 you need to score text describing extremely strange or unnatural situations. Answer only with a number. Here is the text in Italian: "
BODY	"Evaluate how much an Italian text refers to the body and the bodily functions on a scale from 1 to 9. You should evaluate how much the text includes any kind of reference to body parts and to physiological functions or instincts, such as sleeping, eating, having sex. With score 1 you need to score text without any semantic relatedness to the body or the bodily functions. With score 5 you need to score text with a moderate semantic relatedness to the body or the bodily functions. With score 9 you need to score text with an extreme semantic relatedness to the body or the bodily functions. Answer only with a number. Here is the text in Italian: "
INCORPORATION	"Evaluate how much an Italian text relates to any of the following concepts: 'dreams', 'dreaming', 'sleep', 'experiment', 'EEG', 'electroencephalography', 'actigraph', 'actigraphy', 'accelerometer', 'smartwatch', 'recording', 'recorder', 'voice recording'. Provide a score on a scale from 1 to 9. With score 1 you need to score text without any semantic relatedness to these concepts. With score 5 you need to score text with a moderate semantic relatedness to these concepts. With score 9 you need to score text with extreme relatedness to these concepts. Answer only with a number. Here is the text in Italian: "
LIMITATIONS	"Evaluate how much an Italian text includes elements limiting or which might limit the freedom of the characters on a scale from 1 to 9. You should evaluate how much the text describes or refers to elements limiting or which might limit the freedom of the characters either physically or in terms of ethical and moral boundaries. The score concerns the presence of details which might represent an obstacle or a limitation for the characters, such as a blocked road, the feeling of not being able to move, or refraining from doing something because it is not allowed or appropriate in a situation. With score 1 you need to score text without any limitations to the freedom of the characters. With score 5 you need to score text with a moderate number of limitations to the freedom of the characters. With score 9 you need to score text with several limitations to the freedom of the characters. Answer only with a number. Here is the text in Italian: "
THOUGHT	"Evaluate how much an Italian text describes abstracts thoughts or reasoning of the narrator on a scale from 1 to 9. With score 1 you need to score text without any abstract thoughts or reasonings. With score 5 you need to score text with an average number of thoughts or reasonings. With score 9 you need to score text with several descriptions of thoughts or reasonings. Answer only with a number. Here is the text in Italian: "

MOVEMENT	"Evaluate how much an Italian text describes physical movements on a scale from 1 to 9. You should evaluate how much the situations include any kind of physical movement performed by the characters, including the narrator. The scoring concerns movements which can be only performed with the body, such as running, swimming, flying, jumping. With score 1 you need to score text without any semantic relatedness to physical movements. With score 5 you need to score text with a moderate semantic relatedness to physical movements. With score 9 you need to score text with an extreme semantic relatedness to physical movements. Answer only with a number. Here is the text in Italian: "
SETTINGS	"Evaluate how much an Italian text includes changes of the surrounding environment and of the scenery where the events take place on a scale from 1 to 9. You should evaluate how much the text includes moving or displacement of objects and people from one environment or place to another. The scoring also concerns sudden changes from one scenery to another without any explicit description of the displacement. With score 1 you need to score text without any changes of scenery. With score 5 you need to score text with a moderate number of changes of the scenery. With score 9 you need to score text with several changes of the scenery. Answer only with a number. Here is the text in Italian: "
SOCIAL	"Evaluate how much an Italian text describes social interactions on a scale from 1 to 9. You should evaluate how much the situations include any kind of social interaction, either between two people or between more people, such as talking with somebody, yelling at somebody, taking part to an activity with other people. With score 1 you need to score text without any semantic relatedness to social interactions. With score 5 you need to score text with a moderate semantic relatedness to social interactions. With score 9 you need to score text with an extreme semantic relatedness to social interactions. Answer only with a number. Here is the text in Italian: "
SPACE	"Evaluate how much an Italian text describes the surrounding environment and the space where the events take place on a scale from 1 to 9. You should evaluate how much the text includes details regarding the environments and the spaces, such as descriptions of buildings, rooms or landscapes. With score 1 you need to score text without any descriptions of the surrounding environment. With score 5 you need to score text with moderate descriptions of the surrounding environment. With score 9 you need to score text with several descriptions of the surrounding environment. Answer only with a number. Here is the text in Italian: "
TACTILE	"Evaluate how much an Italian text describes tactile experiences on a scale from 1 to 9. You should evaluate how much the situations include the touching, details that can only be perceived through the touch with any body parts, or activities that include touching or brushing. With score 1 you need to score text without any semantic relatedness to tactile experiences. With score 5 you need to score text with a moderate semantic relatedness to tactile experiences. With score 9 you need to score text with extreme semantic relatedness to tactile experiences. Answer only with a number. Here is the text in Italian: "
TIME	"Evaluate how much an Italian text describes the temporal and sequential aspects of the events on a scale from 1 to 9. You should evaluate how much the text includes details regarding the chronological aspects, the duration and the time coordinates of the events. With score 1 you need to score text without any temporal and sequential aspects. With score 5 you need to score text with moderate temporal and sequential aspects. With score 9 you need to score text with several temporal and sequential aspects. Answer only with a number. Here is the text in Italian: "
VALENCE	"Evaluate the emotional valence of an Italian text on a scale from 1 to 9. The scoring is not related to the emotional strength or intensity. The scoring only concerns the subjective perception of positive, negative, or neutral tone. With score 1 you need to score text with extremely negative emotions, such as sadness. With score 5 you need to score text with neutral emotions, neither positive or negative. With score 9 you need to score text with extremely positive emotions such as happiness. Answer only with a number. Here is the text in Italian: "
VISUAL	"Evaluate how much an Italian text describes visual experiences on a scale from 1 to 9. You should evaluate how much the situations include vision, details that can only be perceived through the eyes and the vision, or activities that include vision. With score 1 you need to score text without any semantic relatedness to visual experiences. With score 5 you need to score text with a moderate semantic relatedness to visual experiences. With score 9 you need to score text with extreme semantic relatedness to visual experiences. Answer only with a number. Here is the text in Italian: "

Table S1. AIs' Prompting for the evaluation of semantic dimensions. *In the left column, the dimension labels. In the right column, the prompting used, including definitions, examples and meaning of the Likert Scale values.*

Table S2

Dimension	Full Model adj R^2	p-value Full model	coefficient name	coefficient beta	p-value coefficient	q-value coefficient
AGENTIVITY	0.183	< 0.00001				
			report_type_1:Sex_1	0.33, CI: 0.05 0.61	0.02193	0.49897
AROUSAL	0.102	< 0.00001				
			Sex_1	-0.30, CI: -0.55 -0.06	0.01608	0.10853
			Education	-0.05, CI: -0.09 -0.00	0.03327	0.17966
			WC_BADA	0.53, CI: 0.10 0.96	0.01557	0.10853
			report_type_1:Education	0.05, CI: 0.01 0.09	0.01544	0.10853
			**report_type_1:ATD**	**0.05, CI: 0.02 0.08**	**0.00166**	**0.04492**
AUDITORY	0.047	< 0.00001				
			report_type_1:Age	-0.02, CI: -0.03 -0.00	0.00903	0.24384
			report_type_1:WC_BADA	-0.72, CI: -1.36 -0.09	0.02610	0.35231
BIZARRENESS	0.433	< 0.00001				
			report_type_1	2.85, CI: 0.56 5.14	0.01474	0.07961
			report_type_1:Age	-0.01, CI: -0.03 -0.00	0.03043	0.13692
			**report_type_1:PSQI**	**-0.09, CI: -0.15 -0.03**	**0.00277**	**0.02496**
			**report_type_1:ATD**	**0.07, CI: 0.03 0.10**	**0.00022**	**0.00296**
			**report_type_1:MW**	**0.27, CI: 0.16 0.38**	**< 0.00001**	**0.00005**
			**report_type_1:SCWT**	**-0.03, CI: -0.05 -0.01**	**0.00691**	**0.04665**
BODY	0.076	< 0.00001				
			report_type_1	-3.17, CI: -5.45 -0.90	0.00629	0.08496
			Age	-0.02, CI: -0.03 -0.01	0.00200	0.05396
			STAI	-0.02, CI: -0.03 -0.00	0.02561	0.23052
LIMITATIONS	0.109	< 0.00001				
			Sex_1	-0.41, CI: -0.70 -0.12	0.00503	0.08745
			ATD	-0.04, CI: -0.07 -0.00	0.03834	0.25881
			WC_BADA	0.68, CI: 0.17 1.20	0.00972	0.08745
			report_type_1:ATD	0.05, CI: 0.01 0.09	0.00660	0.08745
THOUGHT	0.352	< 0.00001				
			Sex_1	-0.33, CI: -0.64 -0.01	0.04030	0.13952
			Age	-0.02, CI: -0.03 -0.01	0.00558	0.07526
			STAI	0.02, CI: 0.00 0.04	0.04134	0.13952
			MW	0.15, CI: 0.03 0.27	0.01820	0.13952
			MEQ	-0.02, CI: -0.03 -0.00	0.03820	0.13952
			**WC_BADA**	**0.93, CI: 0.46 1.40**	**0.00012**	**0.00314**
			report_type_1:STAI	-0.02, CI: -0.03 -0.00	0.04995	0.14984
			report_type_1:BSRT	-0.04, CI: -0.08 -0.01	0.02182	0.13952
			report_type_1:MEQ	0.01, CI: 0.00 0.02	0.03483	0.13952
MOVEMENTS	0.079	< 0.00001				
			MEQ	0.02, CI: 0.00 0.03	0.00898	0.08082
			report_type_1:ATD	0.05, CI: 0.02 0.09	0.00386	0.05217

			report_type_1:MW	0.12, CI: 0.00 0.24	0.04276	0.28864
			report_type_1:MEQ	-0.02, CI: -0.03 -0.01	0.00385	0.05217
SETTINGS	0.185	< 0.00001				
			report_type_1:ATD	0.04, CI: 0.01 0.08	0.00482	0.06501
			**report_type_1:MW**	**0.23, CI: 0.14 0.33**	**< 0.00001**	**0.00008**
			report_type_1:VVIQ	0.01, CI: 0.00 0.02	0.04540	0.40857
SOCIAL	0.135	< 0.00001				
			Sex_1	-0.59, CI: -0.98 -0.20	0.00336	0.09082
			SCWT	-0.04, CI: -0.07 -0.01	0.00729	0.09846
			WC_BADA	0.83, CI: 0.12 1.53	0.02145	0.09880
			report_type_1:Sex_1	0.48, CI: 0.07 0.89	0.02172	0.09880
			report_type_1:Age	-0.02, CI: -0.04 -0.00	0.02016	0.09880
			report_type_1:MW	0.17, CI: 0.01 0.33	0.04170	0.14630
			report_type_1:BSRT	0.06, CI: 0.00 0.11	0.04335	0.14630
			report_type_1:WC_BADA	-1.07, CI: -1.99 -0.16	0.02196	0.09880
SPACE	0.217	< 0.00001				
			**report_type_1:ATD**	**0.07, CI: 0.04 0.10**	**< 0.00001**	**0.00017**
			report_type_1:MW	0.11, CI: 0.02 0.21	0.02284	0.26923
			report_type_1:BSRT	0.04, CI: 0.00 0.07	0.02991	0.26923
TACTILE	0.064	< 0.00001				
			MEQ	0.01, CI: 0.00 0.02	0.00256	0.06923
			report_type_1:WC_BADA	0.49, CI: 0.03 0.95	0.03672	0.49573
TIME	0.178	< 0.00001				
			Age	-0.02, CI: -0.03 -0.01	0.00297	0.08019
			VVIQ	0.01, CI: 0.00 0.03	0.01842	0.12436
			report_type_1:ATD	0.05, CI: 0.01 0.08	0.01134	0.10209
			report_type_1:MW	0.15, CI: 0.03 0.26	0.01042	0.10209
VALENCE	0.068	< 0.00001				
			ATD	0.03, CI: 0.00 0.05	0.03019	0.19031
			BSRT	-0.03, CI: -0.06 -0.00	0.02966	0.19031
			VVIQ	0.01, CI: 0.00 0.02	0.04276	0.19031
			report_type_1:PSQI	0.07, CI: 0.02 0.12	0.00727	0.19031
			report_type_1:BSRT	0.04, CI: 0.01 0.07	0.01463	0.19031
			report_type_1:SCWT	0.02, CI: 0.00 0.03	0.03620	0.19031
VISUAL	0.193	< 0.00001				
			Sex_1	-0.36, CI: -0.66 -0.06	0.01991	0.17921
			report_type_1:Sex_1	0.43, CI: 0.12 0.74	0.00712	0.09612
			**report_type_1:ATD**	**0.07, CI: 0.03 0.10**	**0.00079**	**0.02136**

Table S2. Performance measures of the GLME model for the prediction of semantic dimensions based on individual psychological variables in the main dataset. *The GLME model included psychological variables and their interaction with vigilance states (report_type) as regressors of interest and age, sex, education level, and the BADA score as covariates; q<0.05, False discovery Rate -FDR- correction. Significant effects after FDR correction are reported in bold.*

Table S3

	Domain	Full Model adj R^2	p-value Full model	coefficient name	coefficient beta	p-value coefficient	q-value coefficient
	DOMAIN2	0.067	0.00005				
				Sex_1	0.48, CI: 0.07 0.89	0.02106	0.18955
				Age	0.02, CI: 0.00 0.04	0.01312	0.17707
				report_type_1:Sex_1	-0.56, CI: -1.07 -0.06	0.02899	0.19570
				report_type_1:VVIQ	0.03, CI: 0.01 0.05	0.00638	0.17214
	DOMAIN4	0.023	0.00001				
	Food	0.020	0.04967				
				STAI	-0.02, CI: -0.04 -0.00	0.02337	0.31552
				report_type_1:STAI	0.03, CI: 0.00 0.06	0.02041	0.31552
	Objects	0.027	< 0.00001				
				MW	0.20, CI: 0.03 0.36	0.02102	0.18917
				**ROCFr**	**-0.07, CI: -0.11 -0.03**	**0.00034**	**0.00564**
				**report_type_1:ROCFr**	**0.09, CI: 0.04 0.14**	**0.00042**	**0.00564**
	Reactions	0.016	0.34785				
				report_type_1:Sex_1	-0.44, CI: -0.88 -0.01	0.04400	0.59405
				report_type_1:MW	-0.19, CI: -0.36 -0.02	0.02596	0.59405
	DOMAIN12	0.016	0.00026				
				**Age**	**-0.04, CI: -0.06 -0.02**	**0.00064**	**0.01725**
				ROCFr	0.05, CI: 0.00 0.09	0.03856	0.50457
	Matter	0.014	0.00033				
				**PSQI**	**-0.18, CI: -0.30 -0.06**	**0.00245**	**0.03306**
				BSRT	0.07, CI: 0.01 0.13	0.03294	0.25087
				WC_BADA	1.02, CI: 0.06 1.97	0.03717	0.25087
				**report_type_1:PSQI**	**0.22, CI: 0.08 0.36**	**0.00193**	**0.03306**
	Thriller	0.036	< 0.00001				
				Sex_1	-0.42, CI: -0.80 -0.04	0.03022	0.29247
				Age	-0.02, CI: -0.03 -0.00	0.04070	0.29247
				BSRT	0.06, CI: 0.01 0.11	0.02726	0.29247
				report_type_1:Sex_1	0.48, CI: 0.01 0.94	0.04333	0.29247
	Healthcare	0.042	< 0.00001				
				report_type_1:Age	0.02, CI: 0.00 0.04	0.03491	0.47133
				report_type_1:MEQ	-0.02, CI: -0.05 -0.00	0.02742	0.47133
	Locations	0.067	< 0.00001				
				MW	-0.16, CI: -0.30 -0.01	0.03198	0.28783
				BSRT	0.07, CI: 0.02 0.12	0.00499	0.09158
				report_type_1:ATD	0.05, CI: 0.00 0.10	0.04628	0.31236
				report_type_1:MW	0.22, CI: 0.06 0.38	0.00678	0.09158
	Geometry	0.056	< 0.00001				
				**report_type_1:ATD**	**0.09, CI: 0.04 0.15**	**0.00060**	**0.01627**
	Nature	0.036	0.00005				

			MEQ	0.02, CI: 0.00 0.03	0.01670	0.22550
			**report_type_1:ATD**	**0.07, CI: 0.03 0.12**	**0.00176**	**0.04748**
			report_type_1:MEQ	-0.02, CI: -0.03 -0.00	0.04567	0.41107
Fantasy	0.005	0.04715				
			BSRT	0.06, CI: 0.01 0.10	0.02616	0.35309
			report_type_1:Age	-0.03, CI: -0.05 -0.00	0.02398	0.35309
Measurements	0.006	0.01810				
Architecture	0.051	< 0.00001				
Animals	0.080	< 0.00001				
			BSRT	0.08, CI: 0.02 0.14	0.01202	0.32445
Concerns	0.035	< 0.00001				
			Sex_1	0.37, CI: 0.08 0.67	0.01361	0.18374
			STAI	0.02, CI: 0.00 0.04	0.03784	0.34054
			WC_BADA	0.76, CI: 0.17 1.35	0.01129	0.18374
Appearance	0.082	< 0.00001				
			**report_type_1**	**-5.82, CI: -9.87 -1.76**	**0.00494**	**0.04854**
			Sex_1	-0.49, CI: -0.98 -0.01	0.04661	0.17362
			Age	-0.02, CI: -0.05 -0.00	0.01971	0.11271
			**PSQI**	**-0.15, CI: -0.26 -0.05**	**0.00539**	**0.04854**
			MW	0.21, CI: 0.03 0.39	0.02455	0.11271
			report_type_1:Education	0.10, CI: 0.01 0.18	0.02505	0.11271
			**report_type_1:PSQI**	**0.17, CI: 0.06 0.29**	**0.00331**	**0.04854**
Jobs	0.050	< 0.00001				
			Age	0.02, CI: 0.00 0.03	0.00723	0.06508
			report_type_1:Sex_1	0.56, CI: 0.16 0.95	0.00543	0.06508
			**report_type_1:Age**	**-0.03, CI: -0.04 -0.01**	**0.00110**	**0.02973**
			report_type_1:MEQ	-0.02, CI: -0.04 -0.00	0.04027	0.27180
Drama	0.079	< 0.00001				
			Sex_1	-0.42, CI: -0.72 -0.12	0.00653	0.12416
			Age	-0.01, CI: -0.03 -0.00	0.04102	0.22015
			STAI	0.02, CI: 0.00 0.04	0.02903	0.22015
			PSQI	-0.08, CI: -0.15 -0.02	0.00920	0.12416
			WC_BADA	0.60, CI: 0.02 1.18	0.04103	0.22015
			report_type_1:MEQ	0.02, CI: 0.00 0.03	0.04892	0.22015
Society	0.031	0.00010				
			STAI	-0.03, CI: -0.05 -0.01	0.01050	0.09450
			WC_BADA	1.05, CI: 0.35 1.75	0.00318	0.08576
			report_type_1:WC_BADA	-1.22, CI: -2.12 -0.32	0.00796	0.09450
Humanities	0.112	< 0.00001				
			Sex_1	0.42, CI: 0.10 0.73	0.01016	0.09144
			Age	-0.01, CI: -0.03 -0.00	0.04734	0.27520
			report_type_1:Sex_1	-0.66, CI: -1.10 -0.22	0.00312	0.05163
			report_type_1:PSQI	0.13, CI: 0.04 0.22	0.00382	0.05163
Transportation	0.018	0.00216				

Timing	0.179	< 0.00001				
			report_type_1	-3.74, CI: -6.49 -1.00	0.00751	0.10133
			MW	-0.11, CI: -0.22 -0.00	0.04425	0.29869
			report_type_1:Education	0.09, CI: 0.03 0.15	0.00192	0.05195
			report_type_1:ATD	0.05, CI: 0.01 0.09	0.01805	0.16241
Technology	0.023	< 0.00001				
			Education	0.06, CI: 0.01 0.11	0.01440	0.19435
			SCWT	-0.02, CI: -0.04 -0.00	0.04057	0.29351
			WC_BADA	0.61, CI: 0.02 1.21	0.04406	0.29351
			report_type_1:WC_BADA	-1.29, CI: -2.31 -0.26	0.01427	0.19435
Education	0.046	< 0.00001				
			**Age**	**-0.02, CI: -0.04 -0.01**	**0.00022**	**0.00593**
			report_type_1:VVIQ	-0.02, CI: -0.03 -0.00	0.03059	0.41295
Communication	0.035	< 0.00001				
			**Age**	**-0.03, CI: -0.04 -0.01**	**0.00013**	**0.00352**
			MEQ	-0.02, CI: -0.03 -0.00	0.01911	0.17203
			report_type_1:Education	0.07, CI: 0.00 0.13	0.04110	0.27743
			**report_type_1:MEQ**	**0.03, CI: 0.01 0.04**	**0.00353**	**0.04769**
Slang	0.017	< 0.00001				
			**Sex_1**	**0.64, CI: 0.27 1.02**	**0.00085**	**0.01454**
			**Age**	**-0.03, CI: -0.06 -0.01**	**0.00145**	**0.01454**
			PSQI	-0.12, CI: -0.21 -0.03	0.01076	0.07261
			ATD	-0.05, CI: -0.10 -0.01	0.02683	0.14486
			report_type_1:Sex_1	-0.64, CI: -1.24 -0.04	0.03706	0.16678
			**report_type_1:MW**	**-0.39, CI: -0.63 -0.15**	**0.00162**	**0.01454**

Table S3. Performance measures of the GLME model for the prediction of lexical domains based on individual psychological variables in the main dataset. *The GLME model included psychological variables and their interaction with vigilance states (report_type) as regressors of interest and age, sex, education level, and the BADA score as covariates; q<0.05, False discovery Rate -FDR- correction. Significant effects after FDR correction are reported in bold.*

Table S4

Dimension	Full Model adj R^2	p-value Full model	coefficient name	coefficient beta	p-value coefficient	q-value coefficient
AGENTIVITY	0.013	0.11558				
AROUSAL	0.110	0.23799				
			PC4_report	0.07, CI: 0.00 0.13	0.03539	0.60155
AUDITORY	0.050	0.28287				
			Age	-0.01, CI: -0.02 -0.00	0.02824	0.48008
BIZARRENESS	0.169	0.00001				
			Age	-0.02, CI: -0.04 -0.00	0.03236	0.13752
			PSQI	-0.14, CI: -0.23 -0.05	0.00194	0.01652
			MW	0.35, CI: 0.18 0.51	0.00006	0.00094
			SCWT	-0.03, CI: -0.06 -0.00	0.03218	0.13752
BODY	0.034	0.66715				
LIMITATIONS	0.099	0.39999				
THOUGHT	0.177	0.13254				
			WC_BADA	0.92, CI: 0.26 1.59	0.00638	0.10854
MOVEMENTS	0.090	0.02157				
			ATD	0.04, CI: 0.00 0.08	0.03887	0.33043
			PC3_report	0.11, CI: 0.02 0.19	0.01098	0.18659
SETTINGS	0.114	0.00010				
			MW	0.22, CI: 0.09 0.35	0.00112	0.01899
			**PC2_report**	**0.09, CI: 0.03 0.16**	**0.00274**	**0.02325**
SOCIAL	0.076	0.00083				
			Age	-0.03, CI: -0.05 -0.01	0.00026	0.00438
			PC1_report	0.06, CI: 0.01 0.10	0.01041	0.08844
SPACE	0.139	0.00046				
			ATD	0.07, CI: 0.03 0.11	0.00029	0.00496
			BSRT	0.05, CI: 0.01 0.10	0.01950	0.11051
			PC2_report	0.08, CI: 0.02 0.13	0.01182	0.10046
TACTILE	0.051	0.43745				
TIME	0.096	0.04319				
			PC2_report	0.06, CI: 0.01 0.12	0.03168	0.53855
VALENCE	0.031	0.49050				
			PC4_report	-0.05, CI: -0.10 -0.00	0.03326	0.56540
VISUAL	0.122	0.00784				
			ATD	0.07, CI: 0.03 0.12	0.00222	0.03767

Table S4. Performance measures of the GLME model for the prediction of semantic dimensions in dream reports from the main dataset, based on sleep patterns. *The GLME model included all individual predictors that were found to be significant (uncorrected p-value < 0.05) in the previous analysis. For assessing the impact of sleep macrostructure, scores for the four actigraphic PCs were included as regressors of interest; q<0.05, False discovery Rate -FDR- correction. Significant effects for actigraphic PCs after FDR correction are reported in bold.*

Table S5

	Domain	Full Model adj R^2	p-value Full model	coefficient name	coefficient beta	p-value coefficient	q-value coefficient
	DOMAIN2	0.059	0.37824				
	Food	0.010	0.37056				
	Objects	0.006	0.01581				
				MW	0.20, CI: 0.08 0.33	0.00100	0.01699
	Reactions	-0.000	0.45481				
	DOMAIN12	0.010	0.01151				
				Age	-0.03, CI: -0.05 -0.01	0.00061	0.01044
	Matter	-0.002	0.79193				
	Thriller	0.026	0.34324				
	Healthcare	0.007	0.00459				
				Age	0.02, CI: 0.00 0.03	0.04305	0.24398
				MEQ	-0.04, CI: -0.06 -0.02	0.00024	0.00406
				PC4_report	-0.13, CI: -0.26 -0.01	0.03594	0.24398
	Locations	0.038	0.01283				
				ATD	0.04, CI: 0.00 0.08	0.03888	0.16524
				BSRT	0.04, CI: 0.00 0.08	0.03280	0.16524
				PC2_report	0.08, CI: 0.02 0.15	0.01265	0.16524
				PC4_report	-0.09, CI: -0.17 -0.01	0.03643	0.16524
	Geometry	0.034	0.02059				
				ATD	0.05, CI: 0.02 0.09	0.00519	0.08826
				PC3_report	0.09, CI: 0.01 0.17	0.02227	0.18925
	Nature	0.040	0.11205				
				ATD	0.05, CI: 0.01 0.08	0.01303	0.22150
	Fantasy	-0.001	0.56986				
	Animals	0.065	0.80568				
	Concerns	0.001	0.30704				
				PC3_report	-0.10, CI: -0.19 -0.01	0.02545	0.43268
	Appearance	0.075	0.07281				
				Sex	-0.37, CI: -0.74 -0.00	0.04790	0.41799
				PC2_report	0.08, CI: 0.00 0.15	0.04918	0.41799
	Jobs	0.016	0.00160				
				Age	-0.01, CI: -0.02 -0.00	0.00862	0.07325
				PC2_report	-0.08, CI: -0.14 -0.02	0.00763	0.07325
	Drama	0.048	0.00013				
				Sex	-0.64, CI: -0.91 -0.36	< 0.00001	0.00010
				Age	-0.02, CI: -0.03 -0.01	0.00040	0.00336
	Society	0.025	0.29664				
	Humanities	0.016	0.00015				
				Age	-0.03, CI: -0.04 -0.01	0.00105	0.01783
				PSQI	0.08, CI: 0.01 0.14	0.02103	0.17873

	Timing	0.009	0.00717				
				Education	0.05, CI: 0.01 0.09	0.02127	0.18076
				PC2_report	0.06, CI: 0.00 0.12	0.04757	0.26959
				PC3_report	0.10, CI: 0.03 0.17	0.00678	0.11530
	Technology	0.001	0.27806				
	Education	0.012	0.13195				
	Communication	0.003	0.12507				
				MEQ	0.01, CI: 0.00 0.03	0.03817	0.43460
	Slang	0.006	0.02751				
				MW	-0.29, CI: -0.48 -0.10	0.00305	0.05178

Table S5. Performance measures of the GLME model for the prediction of lexical domains in dream reports from the main dataset, based on sleep patterns. *The GLME model included all individual predictors that were found to be significant (uncorrected p-value < 0.05) in the previous analysis. For assessing the impact of sleep macrostructure, scores for the four actigraphic PCs were included as regressors of interest; q<0.05, False discovery Rate -FDR- correction. Significant effects for actigraphic PCs after FDR correction are reported in bold.*

Table S6

Dimension	Full Model adj R^2	p-value Full model	Dataset coefficient	Cohen's d	p-value coefficient	q-value coefficient
AGENTIVITY	0.030	< 0.00001	0.27, CI: 0.00 0.54	0.27	0.04657	0.09313
**AROUSAL**	**0.164**	**< 0.00001**	**0.40, CI: 0.17 0.63**	**0.50**	**0.00060**	**0.00482**
AUDITORY	0.057	< 0.00001	-0.17, CI: -0.45 0.12	-0.19	0.25368	0.36881
BIZARRENESS	0.203	< 0.00001	0.07, CI: -0.26 0.39	-0.02	0.68309	0.78067
**BODY**	**0.054**	**< 0.00001**	**0.34, CI: 0.09 0.60**	**0.36**	**0.00869**	**0.02800**
INCORPORATION	-0.001	0.63139	-0.07, CI: -0.18 0.04	-0.12	0.23750	0.36881
**LIMITATIONS**	**0.143**	**< 0.00001**	**0.43, CI: 0.15 0.71**	**0.46**	**0.00281**	**0.01496**
THOUGHT	0.214	< 0.00001	-0.16, CI: -0.41 0.09	-0.03	0.19926	0.35423
**MOVEMENTS**	**0.110**	**< 0.00001**	**0.41, CI: 0.07 0.74**	**0.35**	**0.01689**	**0.04504**
**SETTINGS**	**0.181**	**< 0.00001**	**0.50, CI: 0.22 0.78**	**0.41**	**0.00053**	**0.00482**
**SOCIAL**	**0.126**	**< 0.00001**	**0.47, CI: 0.12 0.82**	**0.48**	**0.00875**	**0.02800**
SPACE	0.175	< 0.00001	0.02, CI: -0.26 0.29	-0.16	0.91321	0.91321
TACTILE	0.064	< 0.00001	-0.01, CI: -0.23 0.20	-0.10	0.90531	0.91321
TIME	0.172	< 0.00001	0.26, CI: 0.03 0.49	0.30	0.02966	0.06778
VALENCE	0.029	0.06388	-0.06, CI: -0.27 0.15	-0.20	0.58434	0.71919
VISUAL	0.150	< 0.00001	0.17, CI: -0.13 0.46	0.09	0.27661	0.36881

Table S6. Performance measures of the GLME model for the prediction of semantic dimensions across main and lockdown datasets. *The GLME model included sex, age, educational level, and the average word count per participant as covariates of no interest; q<0.05, False discovery Rate -FDR- correction. Significant effects after FDR correction are reported in bold. Cohen's d refers to the effect of the dataset (Lockdown – Main datasets).*

Table S7

Domain	Full Model adj R^2	p-value Full model	Dataset coefficient	Cohen's d	p-value coefficient	q-value coefficient
**DOMAIN2**	**0.120**	**0.00124**	**-3.29, CI: -5.30 -1.28**	**-0.79**	**0.00132**	**0.00325**
DOMAIN3	0.009	0.07131	-0.13, CI: -0.50 0.24	0.15	0.47861	0.54972
DOMAIN4	0.035	< 0.00001	0.11, CI: -0.19 0.41	0.57	0.48100	0.54972
Food	0.019	< 0.00001	-0.32, CI: -0.71 0.07	-0.07	0.11223	0.14963
**Objects**	**0.056**	**< 0.00001**	**-1.86, CI: -2.44 -1.28**	**-0.70**	**< 0.00001**	**< 0.00001**
**Reactions**	**0.023**	**< 0.00001**	**0.74, CI: 0.44 1.04**	**0.93**	**< 0.00001**	**< 0.00001**
DOMAIN12	0.012	0.00110	-0.47, CI: -0.92 -0.01	-0.02	0.04558	0.07293
**Matter**	**0.023**	**< 0.00001**	**-0.78, CI: -1.29 -0.26**	**-0.36**	**0.00326**	**0.00651**
Toponyms	0.069	< 0.00001	-0.32, CI: -0.70 0.05	0.03	0.09397	0.13074
**Thriller**	**0.031**	**< 0.00001**	**-1.22, CI: -1.62 -0.83**	**-0.56**	**< 0.00001**	**< 0.00001**
Healthcare	0.003	0.09021	-0.50, CI: -1.05 0.04	-0.19	0.07140	0.10879
**Locations**	**0.065**	**< 0.00001**	**-0.51, CI: -0.84 -0.18**	**-0.39**	**0.00227**	**0.00484**
**Geometry**	**0.072**	**< 0.00001**	**-0.78, CI: -1.11 -0.45**	**-0.43**	**< 0.00001**	**0.00002**
**Nature**	**0.110**	**< 0.00001**	**0.45, CI: 0.18 0.72**	**0.69**	**0.00115**	**0.00307**
**Fantasy**	**0.014**	**< 0.00001**	**0.56, CI: 0.16 0.95**	**0.50**	**0.00553**	**0.00984**
Measurements	0.002	0.12883	-0.38, CI: -1.00 0.24	-0.29	0.22883	0.28164
**Architecture**	**0.055**	**< 0.00001**	**-1.41, CI: -1.86 -0.96**	**-0.83**	**< 0.00001**	**< 0.00001**
Animals	0.083	< 0.00001	0.09, CI: -0.30 0.47	0.12	0.65985	0.72811
**Concerns**	**0.005**	**0.00718**	**0.42, CI: 0.07 0.77**	**0.18**	**0.01783**	**0.03003**
Appearance	0.100	< 0.00001	-0.03, CI: -0.39 0.34	0.20	0.89127	0.92002
**Jobs**	**0.048**	**< 0.00001**	**0.86, CI: 0.58 1.15**	**0.88**	**< 0.00001**	**< 0.00001**
**Conflicts**	**0.053**	**< 0.00001**	**-1.21, CI: -1.68 -0.75**	**-0.70**	**< 0.00001**	**< 0.00001**
**Drama**	**0.118**	**< 0.00001**	**0.68, CI: 0.35 1.01**	**1.09**	**0.00006**	**0.00020**
Society	0.034	< 0.00001	-0.31, CI: -0.67 0.05	-0.29	0.09249	0.13074
Humanities	0.010	0.00117	-0.32, CI: -0.73 0.09	0.18	0.12528	0.16036
**Transportation**	**0.028**	**< 0.00001**	**-0.58, CI: -0.95 -0.21**	**-0.30**	**0.00197**	**0.00449**
**Timing**	**0.025**	**< 0.00001**	**0.53, CI: 0.25 0.82**	**0.51**	**0.00024**	**0.00071**
Technology	0.004	0.03167	-0.07, CI: -0.53 0.40	0.27	0.77246	0.82396
**Education**	**0.021**	**0.00003**	**0.46, CI: 0.15 0.77**	**0.58**	**0.00360**	**0.00677**
**Transaction**	**0.028**	**< 0.00001**	**-1.19, CI: -1.64 -0.74**	**-0.77**	**< 0.00001**	**< 0.00001**
**Communication**	**0.009**	**0.00131**	**-0.75, CI: -1.13 -0.38**	**-0.55**	**0.00008**	**0.00027**
Slang	0.011	0.00061	-0.02, CI: -0.52 0.47	0.14	0.92780	0.92780

Table S7. Performance measures of the GLME model for the prediction of lexical domains across main and lockdown datasets. *The GLME model included sex, age, educational level, and the average word count per participant as covariates of no interest; q<0.05, False discovery Rate -FDR- correction. Significant effects after FDR correction are reported in bold. Cohen's d refers to the effect of the dataset (lockdown – main datasets).*

Table S8

Dimension	Full Model adj R^2	p-value Full model	coefficient name	coefficient beta	p-value coefficient	q-value coefficient
AGENTIVITY	0.183	< 0.00001				
AROUSAL	0.098	< 0.00001				
			report_type_1:time_ranks	0.44, CI: 0.07 0.81	0.02011	0.06499
			**time_ranks**	**-0.79, CI: -1.17 -0.41**	**0.00005**	**0.00043**
AUDITORY	0.044	< 0.00001				
BIZARRENESS	0.427	< 0.00001				
			**report_type_1:time_ranks**	**-0.74, CI: -1.17 -0.31**	**0.00073**	**0.00956**
BODY	0.076	< 0.00001				
LIMITATIONS	0.109	< 0.00001				
			**time_ranks**	**-1.09, CI: -1.52 -0.65**	**< 0.00001**	**0.00001**
THOUGHT	0.348	< 0.00001				
			**report_type_1:time_ranks**	**0.63, CI: 0.24 1.02**	**0.00157**	**0.00956**
			time_ranks	-0.96, CI: -1.44 -0.48	0.00009	0.00049
MOVEMENTS	0.077	< 0.00001				
			report_type_1:time_ranks	-0.51, CI: -0.97 -0.06	0.02658	0.07087
SETTINGS	0.178	< 0.00001				
			report_type_1:time_ranks	-0.45, CI: -0.83 -0.07	0.02031	0.06499
SOCIAL	0.129	< 0.00001				
SPACE	0.212	< 0.00001				
			report_type_1:time_ranks	-0.41, CI: -0.78 -0.04	0.03169	0.07243
TACTILE	0.063	< 0.00001				
TIME	0.176	< 0.00001				
			**report_type_1:time_ranks**	**0.69, CI: 0.26 1.12**	**0.00179**	**0.00956**
			time_ranks	-0.87, CI: -1.31 -0.42	0.00015	0.00060
VALENCE	0.064	< 0.00001				
			**time_ranks**	**0.59, CI: 0.27 0.91**	**0.00025**	**0.00079**
VISUAL	0.190	< 0.00001				

Table S8. Performance measures of the GLME model for the prediction of semantic dimensions across vigilance states in the main dataset, based on time. *The GLME model included vigilance state (report_type) as a regressor of interest, along with its interaction with time, and age, sex, education level, and the BADA score as covariates; q<0.05, False discovery Rate -FDR- correction. Significant effects after FDR correction are reported in bold.*

Table S9

	Domain	Full Model adj R^2	p-value Full model	coefficient name	coefficient beta	p-value coefficient	q-value coefficient
	DOMAIN2	0.066	< 0.00001				
	DOMAIN4	0.023	< 0.00001				
	Food	0.009	< 0.00001				
				time_ranks	-0.63, CI: -1.08 -0.19	0.00539	0.07280
	Objects	0.049	< 0.00001				
				report_type_1:time_ranks	0.84, CI: 0.01 1.67	0.04712	0.50258
				time_ranks	-0.96, CI: -1.73 -0.20	0.01344	0.08601
	Reactions	0.014	0.57757				
	DOMAIN12	0.015	< 0.00001				
	Matter	0.007	0.00011				
	Thriller	0.029	< 0.00001				
	Healthcare	0.055	< 0.00001				
	Locations	0.061	< 0.00001				
	Geometry	0.051	< 0.00001				
	Nature	0.034	< 0.00001				
	Fantasy	0.001	0.15647				
	Measurements	0.007	0.00004				
	Architecture	0.032	< 0.00001				
	Animals	0.071	< 0.00001				
				time_ranks	-0.76, CI: -1.46 -0.05	0.03499	0.18664
	Concerns	0.035	< 0.00001				
	Appearance	0.074	< 0.00001				
	Jobs	0.049	< 0.00001				
				report_type_1:time_ranks	-0.62, CI: -1.21 -0.04	0.03732	0.50258
				time_ranks	0.64, CI: 0.14 1.14	0.01232	0.08601
	Drama	0.072	< 0.00001				
	Society	0.020	< 0.00001				
				**time_ranks**	**-0.95, CI: -1.43 -0.46**	**0.00012**	**0.00384**
	Humanities	0.098	< 0.00001				
				time_ranks	0.67, CI: 0.18 1.16	0.00683	0.07280
	Transportation	0.019	< 0.00001				
	Timing	0.170	< 0.00001				
				report_type_1:time_ranks	0.60, CI: 0.08 1.12	0.02261	0.50258
	Technology	0.031	< 0.00001				
	Education	0.047	< 0.00001				
	Communication	0.031	< 0.00001				
	Slang	0.015	< 0.00001				

Table S9. Performance measures of the GLME model for the prediction of lexical domains across vigilance states in the main dataset, based on time. *The GLME model included vigilance state (report_type) as a regressor of interest, along with its interaction with time, and age, sex, education level, and the BADA score as covariates; q<0.05, False discovery Rate -FDR- correction. Significant effects after FDR correction are reported in bold.*

Table S10

Anonymization code	Selected Words	Notes
NAME	Andrea	The names are listed in order: three male followed by three female names most commonly used in the year 2000 in the Italian Civil Registry, according to data from the Italian National Institute of Statistics (ISTAT, 2023. Indicatori demografici. *Istituto Nazionale di Statistica*).
	Francesco	
	Matteo	
	Alessia	
	Chiara	
	Martina	
PLACE	Torino	The cities are listed in geographic order: three from northern Italy, three from central Italy, and four from southern Italy, including the islands.
	Milano	
	Trieste	
	Firenze	
	Perugia	
	Roma	
	Napoli	
	Potenza	
	Bari	
	Palermo	
	Cagliari	
INSTITUTION	*fattoria* (farm)	Nouns referring to organization, institutes or services. The nouns are grouped and ordered by economic sector: two from the primary sector, six from the secondary, and six from the tertiary.
	porto (harbor)	
	azienda (company)	
	cantiere (construction site)	
	bottega (workshop)	
	negozio (store)	
	ospedale (hospital)	
	banca (bank)	
	scuola (school)	
	università (university)	
	museo (museum)	
	teatro (theater)	
	biblioteca (library)	
	cinema (cinema)	

Table S10. List of representative words chosen for the replacement of anonymization codes. *The first column lists the codes used to mask sensitive information in verbal reports, referring respectively to individuals, locations, and organizations. The second column presents the representative words selected to approximate the lexical information lost during anonymization. The third column provides additional information about the selected words.*

Table S11

Variable Name	Internal Name	Variable Use	Variable Type	Variable level	Variable Description
AGENTIVITY	ACT	dependent	Likert 1-9, 1: extremely passive, 5: observer, 9: extremely active	report	semantic dimension
AROUSAL	ARO	dependent	Likert 1-9	report	semantic dimension
AUDITORY	AUD	dependent	Likert 1-9	report	semantic dimension
BIZARRENESS	BIZ	dependent	Likert 1-9	report	semantic dimension
BODY	BOD	dependent	Likert 1-9	report	semantic dimension
INCORPORATION	INC	dependent	Likert 1-9	report	semantic dimension
LIMITATIONS	LIM	dependent	Likert 1-9	report	semantic dimension
THOUGHT	THO	dependent	Likert 1-9	report	semantic dimension
MOVEMENTS	MOV	dependent	Likert 1-9	report	semantic dimension
SETTINGS	SET	dependent	Likert 1-9	report	semantic dimension
SOCIAL	SOC	dependent	Likert 1-9	report	semantic dimension
SPACE	SPA	dependent	Likert 1-9	report	semantic dimension
TACTILE	TAC	dependent	Likert 1-9	report	semantic dimension
TIME	TIM	dependent	Likert 1-9	report	semantic dimension
VALENCE	VAL	dependent	Likert 1-9, 1: extremely negative, 5: neutral, 9: extremely positive	report	semantic dimension
VISUAL	VIS	dependent	Likert 1-9	report	semantic dimension
DOMAIN2	DOMAIN2	dependent	Binary 0/1	report	lexical domain
DOMAIN3	DOMAIN3	dependent	Binary 0/1	report	lexical domain
DOMAIN4	DOMAIN4	dependent	Binary 0/1	report	lexical domain
Food	DOMAIN6	dependent	Binary 0/1	report	lexical domain
Objects	DOMAIN9	dependent	Binary 0/1	report	lexical domain
Reactions	DOMAIN10	dependent	Binary 0/1	report	lexical domain
DOMAIN12	DOMAIN12	dependent	Binary 0/1	report	lexical domain
Matter	DOMAIN14	dependent	Binary 0/1	report	lexical domain
Toponyms	DOMAIN15	dependent	Binary 0/1	report	lexical domain
Thriller	DOMAIN16	dependent	Binary 0/1	report	lexical domain
Healthcare	DOMAIN18	dependent	Binary 0/1	report	lexical domain
Locations	DOMAIN19	dependent	Binary 0/1	report	lexical domain
Geometry	DOMAIN20	dependent	Binary 0/1	report	lexical domain
Nature	DOMAIN21	dependent	Binary 0/1	report	lexical domain
Fantasy	DOMAIN27	dependent	Binary 0/1	report	lexical domain
Measurements	DOMAIN29	dependent	Binary 0/1	report	lexical domain
Architecture	DOMAIN32	dependent	Binary 0/1	report	lexical domain
Animals	DOMAIN33	dependent	Binary 0/1	report	lexical domain
Concerns	DOMAIN34	dependent	Binary 0/1	report	lexical domain
Appearance	DOMAIN35	dependent	Binary 0/1	report	lexical domain
Jobs	DOMAIN36	dependent	Binary 0/1	report	lexical domain
Conflicts	DOMAIN38	dependent	Binary 0/1	report	lexical domain
Drama	DOMAIN39	dependent	Binary 0/1	report	lexical domain
Society	DOMAIN41	dependent	Binary 0/1	report	lexical domain
Humanities	DOMAIN42	dependent	Binary 0/1	report	lexical domain
Transportation	DOMAIN44	dependent	Binary 0/1	report	lexical domain
Timing	DOMAIN45	dependent	Binary 0/1	report	lexical domain
Technology	DOMAIN46	dependent	Binary 0/1	report	lexical domain

Education	DOMAIN47	dependent	Binary 0/1	report	lexical domain
Transaction	DOMAIN48	dependent	Binary 0/1	report	lexical domain
Communication	DOMAIN49	dependent	Binary 0/1	report	lexical domain
Slang	DOMAIN50	dependent	Binary 0/1	report	lexical domain
Main dataset	dataset1	N/A	N/A	N/A	Name of the main dataset, collected from 03/2020 to 04/2024
Lockdown dataset	dataset2	N/A	N/A	N/A	Name of the lockdown dataset, collected during in 04/2020
Vigilance States	report_type	independent	Categorical, 0 for wakefulness reports, 1 for dreams	report	encode in dataset1 the difference between dream and wakefulness reports
Experiment	Experiment	independent	Categorical, 0 for the main dataset, 1 for the lockdown dataset	report	main dataset (dataset1) is encoded with '0', lockdown dataset (dataset2) with '1'
Participant	Subj	independent	categorical	subject	ID of each participant, used to model random effects
Time	time_ranks	independent	continuous, range 0..1	report	time of acquisition of reports in dataset 1 since beginning, converted in ranks and normalized to 1
Sex	Sex	independent	Categorical, 0 for female participants, 1 for male participants	subject	biological sex assessed by self-report
Age	Age	independent	continuous, in yrs	subject	chronological age
Education	Education	independent	continuous, in yrs	subject	highest degree obtained
Trait anxiety levels	STAI	independent	continuous	subject	C. D. Spielberger et al., Rev. Interam. Psicol. 5 (1971).
Perceived sleep quality	PSQI	independent	continuous	subject	D. J. Buysse et al., Psychiatry Res. 28, 193–213 (1989).
Attitude towards dreaming	ATD	independent	continuous	subject	K. Bulkeley, M. Schredl, Int. J. Dream Res. 12, 7 (2019).
Mind-Wandering (spontaneous, deliberate)	MW	independent	continuous	subject	K. Christoff et al., Nat. Rev. Neurosci. 17, 718–731 (2016).
Verbal memory	BSRT	independent	continuous	subject	H. Babcock, L. Levy, Test and Manual of Directions; the Revised Examination for the Measurement of Efficiency of Mental Functioning (Stoelting, Wood Dale, IL, US, 1940).
Visuospatial memory	ROCFr	independent	continuous	subject	A. Rey, Arch. Psychol. (Geneve) 28, 215–285 (1941).
Subjective circadian preference	MEQ	independent	continuous	subject	J. A. Horne, O. Ostberg, Int. J. Chronobiol. 4, 97–110 (1976).
Vividness of visual imagery	VVIQ	independent	continuous	subject	D. F. Marks, Br. J. Psychol. 64, 17–24 (1973).
Vulnerability to cognitive interference	SCWT	independent	continuous	subject	F. Scarpina, S. Tagini, Front. Psychol. 8, 557 (2017).
BADA	WC_BADA	independent	continuous	subject	Log10 count of words used in the BADA test
Word Count per participant	WC_subj	independent	continuous	subject	average Log10 count of words across reports of a given participant. It was used when comparing dataset2 with dataset1.
PC#1 from actigraphy	PC1_report	independent	continuous	report	sleep fragmentation - PC1 scores of actigraphic data of the night prior the report
PC#2 from actigraphy	PC2_report	independent	continuous	report	long light sleep - PC2 scores of actigraphic data of the night prior the report
PC#3 from actigraphy	PC3_report	independent	continuous	report	stable advanced sleep - PC3 scores of actigraphic data of the night prior the report
PC#4 from actigraphy	PC4_report	independent	continuous	report	unstable advanced sleep - PC4 scores of actigraphic data of the night prior the report

Table S11. Comprehensive description and naming conventions of all collected variables. *Overview of all collected variables and their naming conventions. The first column lists variable names as referenced in the main text; the second column shows their corresponding internal names used in the repository. Columns 3 to 5 provide information on variable type, scale, and source. The final column includes detailed descriptions and, where applicable, references.*
